# Hemodynamic Factors May Play a Critical Role in Neurological Deterioration Occurring within 72 hrs after Lacunar Stroke

**DOI:** 10.1371/journal.pone.0108395

**Published:** 2014-10-23

**Authors:** Yen-Chu Huang, Yuan-Hsiung Tsai, Jiann-Der Lee, Hsu-Huei Weng, Leng-Chieh Lin, Ya-Hui Lin, Chih-Ying Wu, Ying-Chih Huang, Huan-Lin Hsu, Meng Lee, Hsin-Ta Yang, Chia-Yu Hsu, Yi-Ting Pan, Jen-Tsung Yang

**Affiliations:** 1 Department of Neurology, Chang Gung Memorial Hospital at Chiayi, Chang-Gung University College of Medicine, Chiayi, Taiwan; 2 Department of Diagnostic Radiology, Chang Gung Memorial Hospital at Chiayi, Chang-Gung University College of Medicine, Chiayi,Taiwan; 3 Department of Neurosurgery, Chang Gung Memorial Hospital at Chiayi, Chang-Gung University College of Medicine, Chiayi, Taiwan; 4 Department of Emergency Medicine, Chang Gung Memorial Hospital at Chiayi, Chang-Gung University College of Medicine, Chiayi, Taiwan; Creatis UMR CNRS 5220, France

## Abstract

**Background:**

Whether a perfusion defect exists in lacunar infarct and whether it is related to early neurological deterioration (END) is still under debate. The aim of this study was to evaluate whether END in lacunar infarct is related to a perfusion defect using diffusion-weighted imaging (DWI), diffusion tensor imaging (DTI) and perfusion MR imaging.

**Methods:**

One hundred and forty-one consecutive patients had an MRI scan within 30 hours after onset of symptoms and 43 patients with acute lacunar infarct and classic lacunar syndrome were recruited. The MRI sequences included DWI, DTI and cerebral blood flow (CBF) maps to respectively represent the topographic locations of acute infarcts, the corticospinal tract and perfusion defects. The END was defined in reference to the National Institute of Health Stroke Scale (NIHSS) as an increase ≧2 within 72 hours. Cohen's Kappa coefficient was used to examine the reliability between the 2 image readers. A multivariate logistic regression model was constructed adjusting for baseline variables.

**Results:**

Ten out of the 43 patients had END. Patients having END was significantly associated with lower chances of favorable and good outcomes at 3 months (*p* = 0.01 and *p* = 0.002, respectively). END was predicted when the non-core hypoperfused area overlapped on the corticospinal tract, which is defined as the expected END profile. Cohen's Kappa coefficient between the 2 image readers to define images of expected END profiles was 0.74. In 15 patients with expected END profile, 9 had END clinically, whereas 28 patients had no expected END profile, and only 1 patient had END (*p*<0.0001). After adjusting for sex, the expected END profile was still associated with END (odds ratio, 42.2; *p* = 0.002).

**Conclusion:**

Our study demonstrated that the END in acute lacunar stroke is likely related to the transformation of non-core hypoperfused area into infarction in the anatomy of corticospinal tracts.

## Background and Purpose

Early neurological deterioration (END) in acute stroke often leads to poor functional outcome. Progressive motor deficits are the main manifestations of END in lacunar lacunar infarct [1]. However, there may be different pathologies underlying lacunar infarcts: atherosclerosis or embolism affects larger perforating arterioles while lipohyalinosis affects smaller arterioles [2]. Lacunar stroke caused by atheromatous occlusion at the orifice of large caliber penetrating arteries, termed branch atheromatous disease, is associated with the higher possibility of progressive motor deficit compared to that by lipohyalinosis [3]. The pathophysiology of progression is still incompletely understood and may be related to a hemodynamic defect, extension of thrombosis, excitotoxicity, or inflammation [4].

Although perfusion abnormalities are likely to play a critical role in END in acute stroke [5], there is still uncertainty whether a penumbra exists in lacunar infarct and whether END is related to perfusion deficit. Gerraty et al. observed in a perfusion MRI study that a perfusion defect only existed in striatocapsular artery infarction due to large artery atherosclerosis or embolism but not in lacunar infarcts [6]. However, Yamada et al in a perfusion CT study used cerebral blood flow (CBF), cerebral blood volume, and mean transit time (MTT) to predict the progression of lacunar infarct in the territory of the lenticulostriate artery within 24 hours of onset. Their results showed that lower CBF and higher MTT were related to END, and they hypothesized that hemodynamic insufficiency is related to the progresses of infarction [7]. In another study, perfusion abnormalities (decreased CBF and delayed MTT) were found in two thirds of patients with lacunar infarcts, and those with normal perfusion image were free of deterioration. However, abnormal perfusion image was not significantly related to END [8]. The discrepancy between these studies may be partly due to the small infarct size of lacunar infarct, and the enlarged infarct area may not exactly lead to progressive motor deficit if the corticospinal tract is not involved. This is why END in lacunar infarct has been found to be highly related to the relationship between the location of infarct and corticospinal tract [9], [10].

Alawneh el al. found that the non-symptomatic area in oligemia, which is called non-core–non-penumbral tissue, carried a higher risk of infarct and was associated with the worsening of early neurological deficits [11]. The hypothesis of this study is that END in lacunar infarct is related to the transformation of a perfusion defect into infarction in the area of the corticospinal tract. To confirm this hypothesis, we used diffusion-weighted imaging (DWI) and MR perfusion imaging to define the infarct lesion and perfusion defect, termed non-core hypoperfused area, of each patient. The corticospinal tract was defined by diffusion tensor imaging (DTI), which has been applied with good correlation to trace the corticospinal tract to both clinical motor deficits and the prognosis in patients with lacunar stroke [12], [13].

## Methods

### Patients

This prospective study was conducted from December 2010 to December 2013 and was part of an integrated stroke project at Chang Gung Memorial Hospital. In the integrated stroke project, patients with suspected stroke were selected and underwent MRI within 30 hours after the onset of symptoms. Patients with contraindications to MRI study or gadolinium injection were excluded. Only patients with classic lacunar syndromes and acute lacunar infarct were recruited. All of them had a single visible lesion less than 20 mm in diameter in the territories of penetrating arteries on the DWI. The classic lacunar syndromes included pure motor, ataxic hemiparesis, dysarthria-clumsy hand, pure sensory and sensorimotor lacunar syndromes. All the patients received a standard survey for stroke etiologies, including hematologic and biochemistry tests, electrocardiography and carotid/transcranial Doppler ultrasound. Age, sex, cigarette smoking, hypertension, diabetes mellitus, hypercholesterolemia, atrial fibrillation, history of valvular heart disease, prior coronary artery disease and prior cerebrovascular disease were recorded. All the patients underwent MRA study for both intracranial and extracranial vessels. Patients with large artery stenosis or occlusion or evidence of embolic stroke were excluded. All the recruited patients fulfilled the diagnostic criteria of small vessel disease by TOAST classification [14], with the exception of an infarct upper limit of 20 mm in axial diameter in DWI [15]. We adopted a larger infarct size because it is usually larger in the acute phase in DWI, and the size of 20 mm in diameter was recently proposed for small vessel disease [15], [16]. Although elongated shape of lacunar infarct is more likely due to atheroma, there is still no precise definition for upper limit of its length and it was not excluded from lacunar infarct in our study.

The National Institute of Health Stroke Scale (NIHSS) was used to evaluate the stroke severity daily for three days by the same neurologist or study nurse (Y.C.K.). Although END usually refers to deterioration of NIHSS by ≧4 points during the first 48 or 72 hours after onset of stroke [5], [17], [18], another definition by ≧2 points has also been recommended because death and major disability increased among patients with deterioration by this definition [19]. In addition, the END in lacunar stroke is mainly associated with motor deficits [1]. Therefore, END in our study was defined as NIHSS increase ≧2 within 72 hours. The modified Rankin Scale (mRS) at 3 months was evaluated by the study nurse (Y.C.K.). Good outcome was defined as an mRS score of 2 or less, and favorable outcome was defined as an mRS score of 0 or 1.

The study was performed under a protocol approved by the Institutional Review Board of Chang Gung Memorial Hospital, and all examinations were performed after obtaining written informed consent.

### Imaging

All data were collected using a 3 Tesla Siemens Verio MRI system (Siemens Medical System, Erlangen, Germany) using a 16 channel head coil. The MRI protocol, including axial T1- and T2 images, axial DWI, MR angiography, and dynamic susceptibility contrast perfusion imaging, were acquired as described previously [20]. DTI were obtained using a single-shot spin-echo echoplanar imaging sequence (repetition time = 9,300 ms, echo time = 100 ms, field of view = 220 mm, matrix = 88×88, 64×2.5 mm slices, b value = 1,000 s/mm^2^, 30 directions). DSI-Studio software (http://dsi-studio.labsolver.org/) was used in the reconstruction of the corticospinal tract. The corticospinal tract was reconstructed with the seed region of interest (ROI) being on the pontomedullary junction and the target ROI being the anterior midpons. Fiber tracts passing through both ROIs were designated as the corticospinal tract. A fractional anisotropy (FA) threshold of>0.2 and direction threshold <60° were used for the performance of fiber tracking. Maps of CBF using block singular value decomposition in MR perfusion imaging was used to depict the perfusion defect. DWI, tractography and CBF maps were generated for each patient to respectively represent the topographic locations of acute infarcts, the corticospinal tract and perfusion defects.

All patients were classified into four types according to the topographic correlation: type A was defined as no perfusion defect; type B was defined as a CBF perfusion defect within the DWI lesion, indicating no non-core hypoperfused area; types C and D were defined as a CBF perfusion defect beyond the DWI lesion, indicating an existing non-core hypoperfused area. The non-core hypoperfused area did not overlap on the corticospinal tract in type C but overlapped on the corticospinal tract in type D. For this reason, only type D was the expected END profile and was assumed to correlate to END clinically. All the imaging data were evaluated by an experienced stroke neurologist (Y.C.H.) and a neuroradiologist (Y.H.T.), both blinded to the clinical information. The two image readers discussed to reach a consensus if there was a discrepancy of the expected END profile.

On CBF maps, the hypoperfused area was selected visually as ROI 1, as this was applied to judge the image type as well. The ROI 2 was set in the mirror position with same shape. The CBF ratio was defined as (CBF value on ROI 1)/(CBF ratio on ROI 2), representing the degree of hemodynamic insufficiency.

### Statistical analysis

Statistical analyses were performed using the Statistical Program for Social Sciences (SPSS) statistical software (version 18, Chicago, IL, USA). Cohen's Kappa coefficient was used to examine the reliability between the 2 image readers (Y.C.H. and Y.H.T.). Continuous variables were expressed as means ± SD, and comparisons between the two groups were performed using Student's t-test after testing normality with the Kolmogorov-Smirnov test. Fisher's exact test was used for categorical variables. To evaluate the independent effect of image profile in predicting END, a multivariate logistic regression model was constructed adjusting for baseline variables when a *p*-value<0.1 was found in the univariate analysis. All *p*-values were two-tailed. The values of *p*<0.05 were considered significant.

## Results

One hundred and sixty-three consecutive patients had an MRI scan and 42 patients were excluded because of incomplete imaging data for 25 patients and non-stroke or transient ischemic stroke for 17 other patients. A total of 121 patients underwent complete MRI and were diagnosed with acute ischemic stroke. Forty-three patients fulfilled the criteria of small vessel disease and were enrolled in this study (17 women, 26 men; mean age of 69.2±10.3 years). Nineteen patients (44.2%) were pure motor lacunar syndrome, 8 patients (18.6%) were ataxic hemiparesis, 8 patients (18.6%) were dysarthria-clumsy hand, 4 patients (9.3%) were pure sensory and 4 patients (9.3%) were sensorimotor.

All the patients did not receive t-PA treatment and took aspirin once arriving at the emergency department. The NIHSS at admission was 3.6±1.7 and the initial infarct size in DWI was 1.18±1.13 cm^3^. Ten out of 43 patients had END and the time from stroke onset to END was 21.5±15.7 hrs. The demographic data of these patients are shown in Table 1 and the nature of END of each patient is presented in Table 2. All but one patient had END associated with limb weakness, while she was associated with ataxia. Among patients with or without END, only the female gender was more prevalent in END (p = 0.03). Conversely, the factors of age, initial stroke severity, initial infarct size or stroke risk showed no statistically significant difference. Patients with END were significantly associated with lower chances of favorable and good outcomes at 3 months (p = 0.01 and p = 0.002, respectively).

**Table 1:** Demographic and clinical data of enrolled subjects.

	END (n = 10)	No END (n = 33)	*p*
Gender (F/M)	7/3	10/23	0.03*
Age	69.5±11.6	69.0±10.1	0.90
NIHSS	3.2±1.4	3.7±1.8	0.46
Stroke onset to MRI (hr)	13.8±8.1	17.6±7.3	0.23
DWI volume (ml)	1.07±0.54	1.22±1.26	0.58
Diabetes mellitus	6 (60.0%)	16 (48.5%)	0.72
Hypertension	10 (100%)	31 (93.9%)	1.0
Smoking	1 (10%)	12 (36.4%)	0.24
Hyperlipidemia	5 (50.0%)	12 (36.4%)	0.44
Atrial fibrillation	0 (0%)	0 (0%)	-
Old stroke or TIA	2(20.0%)	7 (21.2%)	1.0
Coronary artery disease	0 (0%)	1 (3.0%)	1.0
Systolic BP (mmHg)	184.4±45.8	174.7±31.6	0.45
Diastolic BP (mmHg)	102.9±24.8	102.2±20.6	0.93
Favorable outcome at 3 months	4 (40%)	28 (84.8%)	0.01*
Good outcome at 3 months	4 (40%)	30 (80.9%)	0.002*

TIA = transient ischemic attack; NIHSS = National Institutes of Health Strokes Scale; BP = blood pressure.

*: p<0.05.

**Table 2:** The nature of early neurological deterioration of each patient.

Patient	Initial lacunar syndrome	Early neurological deterioration			
		Facial palsy	Limb weakness	Ataxia	Sensory deficits
1	Pure sensory			Y*	
2	Pure motor	Y	Y		
3	Pure motor		Y		
4	Pure motor		Y		
5	Ataxic hemiparesis	Y*	Y		
6	Ataxic hemiparesis		Y*		
7	Pure motor	Y*	Y		
8	Dysarthria-clumsy hand	Y	Y		
9	Pure motor		Y		Y*
10	Ataxic hemiparesis		Y		

*: new signs of early neurological deterioration.

**Figure 1:**
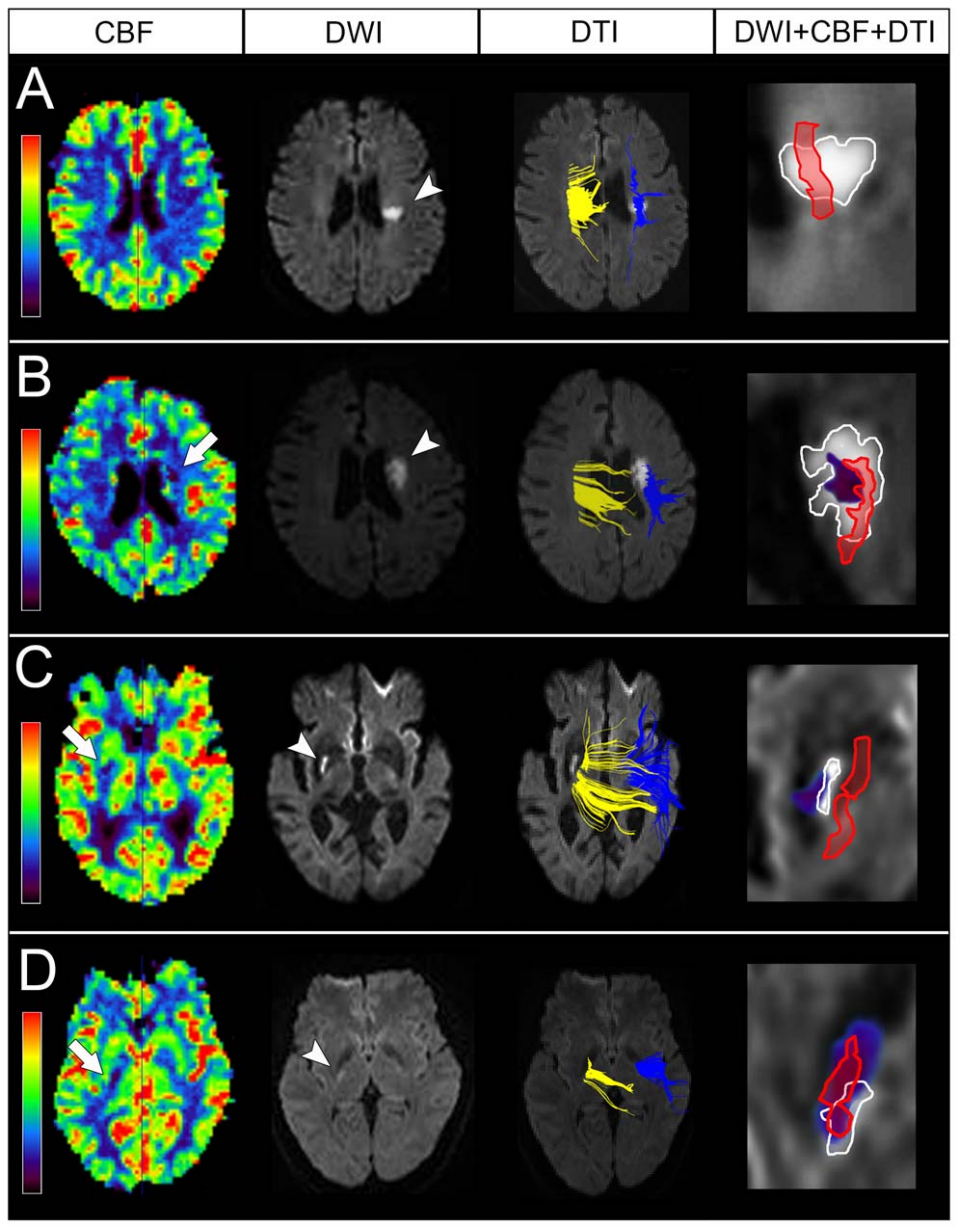
Representative CBF, DWI, DTI and overlapped (DWI+CBF+DTI) maps for each type. The perfusion defects are shown in the CBF maps (arrow) and acute infarcts are shown in DWI maps (arrowhead). DTI maps show right (yellow) and left (blue) corticospinal tracts overlaid on DWI, which is rotated to show the anatomical correlation between acute infarcts and the corticospinal tracts. In the overlapped maps, the regions in white represent acute infarct in DWI, the regions in red represent the corticospinal tract, and the regions in purple represents perfusion defects. Type A is defined as no perfusion defect (A). Type B is defined as a CBF perfusion defect within the DWI lesion, indicating no non-core hypoperfused area (B). The non-core hypoperfused area is defined as a perfusion defect area beyond the DWI lesion. The non-core hypoperfused area is not involved the corticospinal tract in type C (C), and it overlaps on the corticospinal tract in type D (D), which is the expected END profile.

The Cohen's Kappa coefficient between the 2 image readers (Y.C.H. and Y.H.T.) to define the predicted END profile was 0.74, indicating a good interobserver reliability. The represented imaging type is shown in Figure 1. There were 14, 8, 6 and 15 patients classified as types A, B, C, and D, respectively. There were 29 (67.4%) patients who had perfusion defects (types B, C and D) and 21 (48.8%) patients had non-core hypoperfused area (Types C and D). In 15 patients with the expected END profile, 9 had END clinically, whereas 28 patients did not have the expected END profile, and only 1 patient had END (Fisher's exact test: *p*<0.0001; odds ratio: 40.5; 95% confidence interval: 4.3–383.3; Table 3). After adjusting for sex in the multivariate logistic regression model, the expected END profile was still associated with END (*p* = 0.002; odds ratio: 42.2; 95% CI: 3.9–452.9).

**Table 3:** Expected END profile to predict END.

	END (+)	END (−)
Expected END profile (+)	9	6
Expected END profile (−)	1	27

Expected END profile: an existing penumbra with corticospinal tract involvement.

The mean CBF ratio (ROI 1/ROI 2) in the 29 patients with perfusion defects was 0.58, which may suggest that the selected hypoperfused area included both penumbra and oligemia. The mean CBF ratio was not different between the patients with or without END (0.54 vs. 0.60, *p* = 0.37). The mean volumes of perfusion defects were not different between patients with or without END (0.77 vs. 1.16 ml; *p* = 0.07).

## Discussion

Although hemodynamic abnormalities play a critical role in END in acute stroke, there has to now been no direct evidence to prove this association in lacunar stroke. Our study's findings from DTI, DWI and perfusion MR imaging marks the first demonstration that END in acute lacunar stroke is likely related to the transformation of a non-core hypoperfused area into infarction in the anatomy of corticospinal tracts, indicating the existence of penumbra and oligemia. This result supports the hypothesis that END in lacunar infarct is due to hemodynamic compromise.

Lacunar stroke was first proposed by Fisher [2], who observed lacunes in the deep brain structures after occlusion of penetrating arteries, with underlying mechanisms of lipohyalinosis, microatheroma or embolism. Nevertheless, whether the perfusion defect exists in lacunar infarct remains unverified. Only in Gerraty et al's study has the perfusion defect been observed in the larger infarct size by striatocapsular artery occlusion rather than lacunar infarct [6], and this seems to suggest that the perfusion defect was caused to exist in the infarction by microatheroma. However, in our study, there were 29 patients who had perfusion defects and 21 who had non-core hypoperfused area. The major reasons for these discrepancies are that only 6 patients with lacunar infarct were selected in Gerraty et al's study and their infarct volumes were smaller (all<1.1 cm^3^) than those in our study. In addition, higher magnetic field strengths and novel analytic methods for perfusion imaging in our study may have detected these tiny perfusion defects. The perfusion defects were easily observed having large infarct size, which was probably due to microatheroma in the larger penetrating artery. However, we still observed some patients with tiny perfusion defects and acute infarcts less than 10 mm in diameter (Figure 1, D). This suggests that the perfusion defect may also exist in smaller penetrating arteriole occlusions due to lipohyalinosis.

The findings of our study are compatible with Yamada et al.'s perfusion CT study that postulated that hemodynamic insufficiency may be the major cause for the progresses of lacunar infarction [7]. However, the perfusion CT could not identify the acute infarct core and excluded it from the selected ROI. It is thus difficult to prove that the progression of lacunar infarct is related to the enlarged infarct into the non-core hypoperfused area, which would indicate the existence of penumbra. Additionally, the END in their study is defined as occurring within 7 days, which is not really early for hemodynamic insufficiency.

Although some studies suggest END in lacunar stroke is related to larger infarct size due to occlusion of microatheroma [10], [21], [22], the acute infarct size in our study was not different between patients with or without END. However, the expected END profile (existence of non-core hypoperfused area with the corticospinal tract involvement) was an independent predictor for END in our study. This may hint at both infarct location and existing non-core hypoperfused area being important in predicting END. This result is compatible with a previous study that found that infarct location was the only independent predictor for END [9]. This may be why Poppe et al's study failed to predict END by abnormal MR perfusion without the relationship of the corticospinal tract and perfusion defects [8].

Some studies suggest that lacunar infarct is less susceptible to reperfusion treatment due to the absence of existing lysible clots and penumbra [23], [24], [25]. However, a further study found that lacunar infarct was related to better outcomes than other stroke subtypes after thrombolytic therapy, even after adjustment for baseline variables [26]. Our study supports the hypothesis of existing hypoperfused area in lacunar infarcts. In addition, lacunar infarcts with occlusion by atheroma and embolism may be susceptible to thrombolytic therapy. Therefore, thrombolytic therapy may be beneficial in patients with lacunar stroke and existing perfusion defects.

Our study sheds light on predicting END in lacunar stroke. According to our findings, lacunar stroke with non-core hypoperfused area is more likely related to END if the corticospinal tract is involved. With further reference to the anatomic location of the corticospinal tract by DTI, it will be possible to even more accurately predict who will experience END. Because progressive motor deficits in lacunar infarct usually lead to severe disability, patients with high risk of END deserve more aggressive treatments, such as anticoagulants [27] or dual antiplatelet therapy [28], [29]. However, whether the intensive treatments can reduce the risk of END in these patients still needs to be corroborated with future studies.

A potential limitation of this study is that there was no specified threshold to define perfusion deficit in lacunar infarct. Although time to max (Tmax) is widely adopted to define penumbra, it was found to be less sensitive to detect the hypoperfused area than CBF in our cases of lacunar infarcts, probably because the time curve may not have been delayed in the territory of single perforating artery. It is necessary to validate and precisely define the threshold of penumbra or severe oligemia with risk of infarct, as well as to evaluate which MRI parameter is adequate. Moreover, since the perfusion defect was small, there may be some difficulties to determine whether a non-core hypoperfused area overlapped on the corticospinal tract, especially when judged visually. In addition, the time from stroke onset to MRI scan was up to 30 hours in some patients. The perfusion defect may disappear over time and the END may not really be caused by hemodynamic insufficiency. Furthermore, since the hemodynamic change following a stroke event is a dynamic process, to predict END with a single MRI scan may oversimplify the underlying pathophysiology. Hence, a more rigorous test is necessary for this concept especially as applied to clinical practice.
